# Monocyte Function in Parkinson's Disease and the Impact of Autologous Serum on Phagocytosis

**DOI:** 10.3389/fneur.2018.00870

**Published:** 2018-10-16

**Authors:** Ruwani S. Wijeyekoon, Deborah Kronenberg-Versteeg, Kirsten M. Scott, Shaista Hayat, Joanne L. Jones, Menna R. Clatworthy, R. Andres Floto, Roger A. Barker, Caroline H. Williams-Gray

**Affiliations:** ^1^John van Geest Centre for Brain Repair, Department of Clinical Neurosciences, University of Cambridge, Cambridge, United Kingdom; ^2^Wellcome Trust-MRC Cambridge Stem Cell Institute, University of Cambridge, Cambridge, United Kingdom; ^3^Department of Clinical Neurosciences, University of Cambridge, Cambridge, United Kingdom; ^4^Department of Medicine, University of Cambridge, Cambridge, United Kingdom

**Keywords:** Parkinson's, monocyte, phagocytosis, migration, cytokine, serum

## Abstract

**Background:** Increasing evidence implicates involvement of the innate immune system in the initiation and progression of Parkinson's disease (PD). Monocytes and monocyte-derived cells perform a number of functions, such as phagocytosis, chemotaxis, and cytokine secretion, which may be particularly relevant to PD pathology. The behavior of these cells in early-moderate disease, in conditions more similar to the *in-vivo* environment has not been fully evaluated.

**Research Question:** Does monocyte function, including phagocytosis, chemotaxis and cytokine secretion, differ in early-moderate PD compared to age and gender-matched controls?

**Methods:** Participants included PD patients (*n* = 41) with early-moderate stage disease (Hoehn and Yahr ≤2) and age and gender matched controls (*n* = 41). Peripheral blood mononuclear cells (PBMCs) were isolated from whole blood and monocytes were further separated using CD14 magnetic beads. Functional assays, including bead phagocytosis (in standard medium and autologous serum), Boyden chamber trans-well chemotaxis, and cytokine secretion on lipopolysaccharide stimulation were performed. Monocyte surface markers relating to chemotaxis were measured using immunohistochemistry and flow cytometry. Between-group analysis was performed using paired *t*-tests.

**Results:** An autologous serum environment significantly increased bead phagocytosis compared to standard medium as expected, in both patients and controls. When in autologous serum, PD monocytes demonstrated enhanced phagocytosis compared to control monocytes (*p* = 0.029). The level of serum-based phagocytosis was influenced by complement inactivation and the origin of the serum. There were no significant differences between PD and controls in terms of standard medium based monocyte migration or cytokine secretion in this cohort.

**Conclusions:** Autologous serum has a significant influence on monocyte phagocytosis and reveals increased phagocytic capacity in early-moderate PD compared to controls. These conditions may better reflect the function of monocytes *in-vivo* in PD patients than standard medium based phagocytosis assays. Further studies will be required to replicate these results in larger cohorts, including earlier and later stages of disease, and to understand which serum factors are responsible for this observation and the potential mechanistic relevance to PD pathogenesis.

## Introduction

There is increasing evidence of an association between the innate immune system and Parkinson's disease (PD), with genetic, cellular and biomarker studies suggesting variations in innate immune genes and cells in this condition (1). A few studies have found peripheral innate immune changes in PD patients, including changes in peripheral monocyte phenotype and function (2–4), but results are inconsistent between studies and may be related to variations in participant characteristics and assay protocols.

Monocytes are a major component of the peripheral innate immune system and have a number of important functions, such as migration into tissues, phagocytosis of pathogens and cell debris, secretion of cytokines and other proteins, antigen presentation to cells of the adaptive immune system and differentiation into dendritic cells and macrophages which take on more specialized roles (5). The 3 principal subtypes of monocyte–classical, intermediate and non-classical-specialize in different combinations of functions. However, there is a considerable degree of overlap, and variability between study findings suggests that the functional abilities of monocytes may vary depending on a variety of intrinsic and extrinsic factors (5–8).

With relevance to PD pathology, monocytes and related cells, such as macrophages have been found to be capable of entering and interacting with the central nervous system via the meninges and choroid plexus (9, 10) and may be involved in the recognition and/or phagocytosis of protein aggregates (11–13), of debris from degenerating neurons and other cells, or of microbial organisms and their components. However, this may not be entirely beneficial, as they may also generate inflammatory cytokines, such as IL-1β in response to TLR and inflammasome stimulation by forms of alpha-synuclein or other pathogen or damage associated molecular patterns (PAMPs and DAMPs) (12), which in turn may then drive an ongoing inflammatory response. Monocyte derived cells may also conceivably play a role in the presentation of pathogenic alpha-synuclein and other antigens, to T lymphocytes, mediated by the PD-associated risk allele HLA-DR (14–16). These functions may occur within the intravascular compartment, or monocytes could also migrate into tissues to function as monocytes or differentiated cells, such as dendritic cells and macrophages (15).

Studies on peripheral monocyte function in PD to date have reported impaired phagocytosis, with higher monocyte alpha-synuclein levels associated with greater phagocytic impairment (2, 3). Uptake of alpha-synuclein fibrils by monocytes (12) has been shown to increase production of pro-inflammatory cytokines, such as IL-1β, while monocyte uptake of alpha-synuclein oligomers has been reported to decrease with age (11). Monocyte cytokine secretion in response to stimulation has been found to be increased (3, 17) or decreased (4) in PD compared to controls in separate studies. No studies have assessed monocyte migration in PD, but alpha-synuclein monomers and oligomers have been found to act as monocyte chemotactic factors (18).

While studies to date have not been entirely consistent, they have provided some indication that there may be differences in monocyte function between PD and controls. However, most studies have been performed on relatively late stage PD patients with average disease duration of at least 8–10 years, in whom secondary effects of the disease process on the innate immune system are likely to be predominant. Functional differences in monocytes of PD patients at an earlier disease duration and stage than in these previous studies, have not been fully assessed.

Functions, such as phagocytosis may also be affected by changes in cell surface marker expression and properties, which occur on prolonged incubation in different forms of artificial media (19). Thus, assessment of functional phagocytic differences in conditions which more closely replicate the *in-vivo* state, with less pre-incubation and an autologous serum environment, may also be important in order to obtain a better understanding of monocyte function in PD.

Therefore, this study investigated key functions of *ex-vivo* monocytes (phagocytosis, migration and cytokine secretion), in early-moderate PD patients [Hoehn and Yahr (HY) stage ≤2, mean disease duration 4.2 ± 1.1 years] and age and gender matched controls, using conditions more representative of the *in-vivo* state where possible. Monocyte migration-related markers [CCR2, C-X3-C motif chemokine receptor 1 (CX3CR1)] were also evaluated using immunocytochemistry and flow cytometry.

## Materials and methods

### Participant recruitment and sample collection

The study was carried out and the protocol was approved in accordance with the recommendations of the Cambridgeshire Research Ethics Committee (03/303), with written, informed consent from all subjects in accordance with the Declaration of Helsinki. Patients were recruited from the PD Research Clinic at the John van Geest Centre for Brain Repair in Cambridge.

Inclusion criteria were fulfillment of UK PD Brain Bank Criteria for a diagnosis of PD, age 55–80 years and Hoehn and Yahr (HY) stage ≤2 as defined by the Movement Disorder Society, with absence of postural instability (20). Exclusion criteria were: other neurodegenerative disorders, chronic inflammatory or autoimmune disorders, current clinically significant infection, surgery within last month, vaccinations in the last 3 weeks, use of anti-inflammatory/immunomodulatory medications [steroids (within 3 months), high dose aspirin >75 mg (2 weeks), ibuprofen and other nonsteroidal anti-inflammatory drugs (2 weeks) and other long-term immunosuppressant drugs e.g., azathioprine, mycophenolate, methotrexate, rituximab or other antibody therapy (1 year)].

Control participants were recruited from the NIHR Cambridge BioResource (http://www.cambridgebioresource.org.uk). They were age and gender matched to the patients and had no history of neurological disease, self-reported memory problems or depression. Exclusion criteria for controls were the same as for the patients.

50 ml venous blood was collected (45 ml lithium heparin and 5 ml serum in Sarstedt, S-Monovette® tubes) between 9 and 11 a.m. and patients were on their regular medication and had no dietary restrictions. Serum was extracted by centrifuging samples at 2,000 rpm for 15 min, following 15 min clotting time. Separated serum was stored at 4°C prior to subsequent processing. PBMCs were isolated for immunohistochemistry and flow cytometry. Functional assays were performed on fresh cells and serum, depending on cell availability. Patient and paired control samples were processed together on the same day.

Basic demographic and clinical data were obtained from the patients and included disease duration, medication history, Unified Parkinson's Disease Rating Scale (UPDRS) score and Addenbrooke's Cognitive Examination-Revised (ACE-R) score.

Separate data from this participant cohort contributed toward our previously published study investigating T cell senescence in PD (21).

### PBMC isolation, immunocytochemistry and flow cytometry

PBMCs were extracted using the standard Ficoll gradient centrifugation method (Ficoll® Paque Plus, GE Healthcare). Cell suspensions were centrifuged, and cell pellets were blocked with fluorescence activated cell sorting (FACS) buffer with 2% mouse serum (Sigma) per 0.5–1 × 10^6^ cells. Following blocking for 30 min, the PBMCs were stained with a panel of relevant conjugated antibodies including [CX3CR1-APC and CCR2-PE (Biolegend)] or appropriate isotype controls [Rat IgG2b κ-APC and Mouse IgG2a κ-PE (Biolegend)] and incubated at 4°C for 30 min. Following incubation, the PBMCs were washed and then fixed with 2% paraformaldehyde (PFA) and re-suspended in FACS buffer for flow cytometry. Flow cytometry was performed using the BD LSR Fortessa machine with BD FACS Diva software.

Monocytes were gated as described in the literature (22) (Supplementary Figure 4) and a minimum number of 10,000 monocyte events were collected per sample. PBMCs from healthy controls, labeled with single conjugated antibodies, were used to determine the appropriate compensation for spectral overlap of fluorophores.

Flow cytometry data was analyzed using Flow Jo software, version 10. The percentage of positive cells and marker expression levels were determined with reference to isotype control samples (Median Fluorescence Intensity (MFI) Test/Isotype ratio).

### Monocyte separation

CD14^+^ cells were separated using MACS® magnetic CD14^+^ beads (Miltenyi Biotec) and “LS” columns, according to the manufacturer's instructions. Patient and control pairs were separated using the same method. CD14^+^ cell purity following magnetic bead separation was >97% (CD14-APC-H7, Biolegend; Supplementary Figure 1A). The monocytes obtained following CD14^+^ magnetic bead separation undertaken in standard cooled conditions produced consistent patterns of CD14/CD16 staining, demonstrating the presence of all monocyte subtypes (Classical, Intermediate and Non-Classical; Supplementary Figure 1B).

### Monocyte bead phagocytosis assays

CD14^+^ cells were centrifuged at 350 g for 5 min and re-suspended in either phenol-red free, clear RPMI (Life Technologies) + 10% heat inactivated FCS (Sigma), or in 100% of the participant's own serum (200 μl per 0.5 × 10^6^ monocytes). The cells were placed in 96 well-plates at a density of 0.5 × 10^6^ monocytes in 200 ul per well and equilibrated in the incubator (37°C, 5% CO_2_; Test plates) or the fridge (4°C plates) for 45 min.

**Figure 1:**
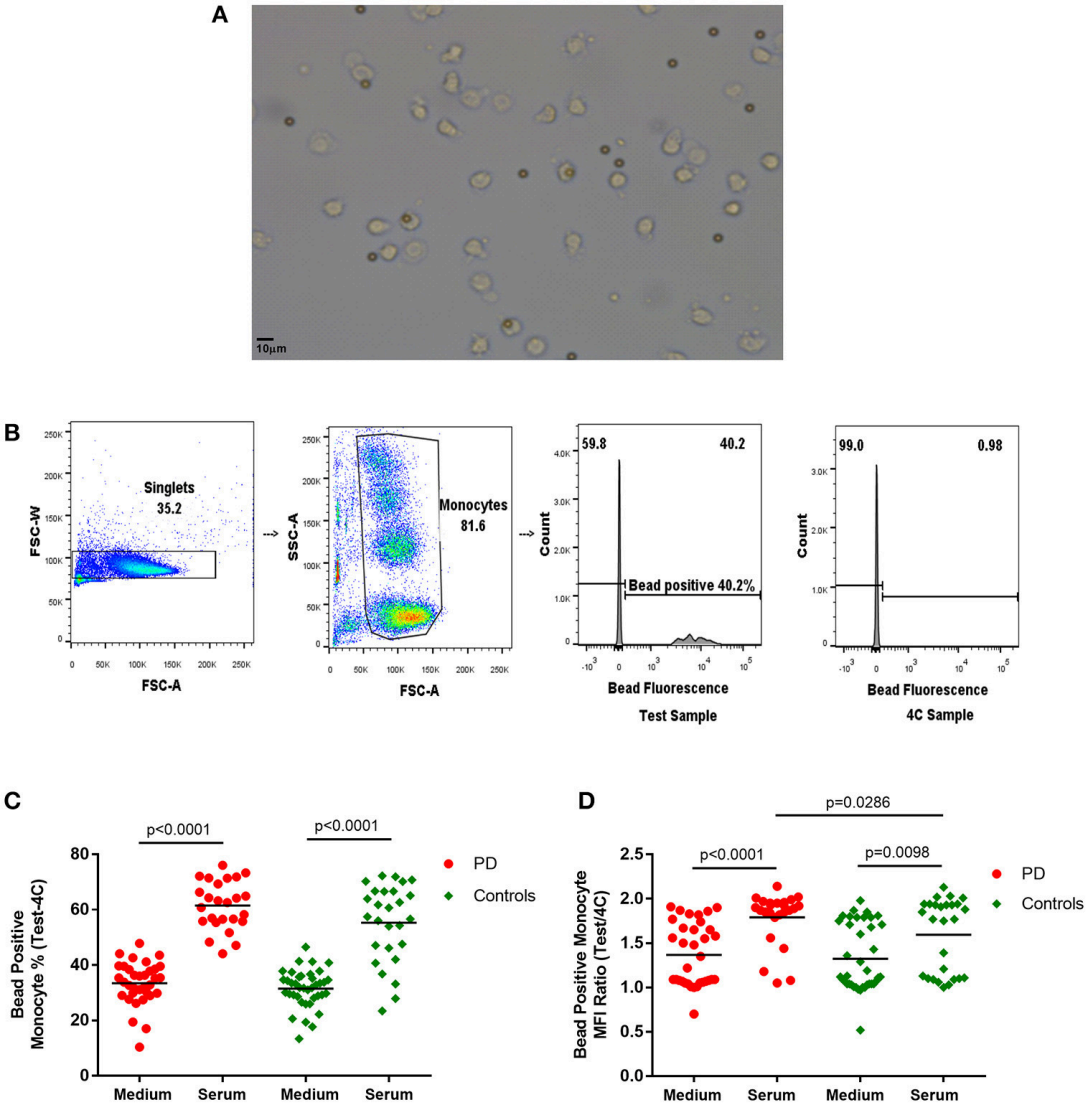
**(A)** Light microscope image of monocytes with phagocytosed 2–5 μm latex beads (× 20). **(B)** Flow cytometry gating strategy for monocyte latex bead phagocytosis (1:1 ratio) analysis. Monocyte gate extended upwards to include bead phagocytosed monocytes, which have increased side scatter. Dividing gate on histogram based on position of 4°C sample. FSC-A, forward scatter-area; FSC-W, forward scatter-width; SSC-A, side scatter-area. **(C,D)** Monocyte latex bead phagocytosis in standard medium and in autologous serum–**(C)** percentage bead positive monocytes (37–4°C); **(D)** bead positive monocyte median fluorescence intensity (MFI) ratio (37/4°C) [Medium-PD = 34, Controls = 39; Serum-PD = 25, Controls = 27; *p*-values relate to the significance of paired *t*-tests performed between matched PD and control pairs following the exclusion of experimental outliers >3 SD above or below mean (excluded pairs Medium = 1, Serum = 0)].

Latex beads [Fluorescent Carboxyl Polymer, Dragon green, 2–5 μm (Bangs Laboratories)] were added at a 1:1 ratio and mixed into the appropriate wells. The Test plates were placed in the incubator (37°C, 5% CO_2_) to simulate *in-vivo* conditions. Reference plates were placed in the fridge (4°C) to inhibit monocyte phagocytosis and endocytosis and used as a reference to account for non-specific adherence of fluorescent beads to the cells for each condition. Cells were incubated for 60 min, then washed with ice cold PBS and then with FACS buffer. Cells were fixed in 2% PFA prior to flow cytometry (BD LSR Fortessa). The gating strategy is illustrated in Figure 1. The bead positive monocyte percentage and bead positive monocyte MFI ratio values for bead uptake were calculated with reference to the 4°C samples (Bead positive monocyte % = Test sample bead positive monocyte %−4C sample bead positive monocyte %; Bead positive monocyte MFI ratio = Test sample bead positive MFI/4C sample bead positive MFI).

The effect of heat mediated serum complement inactivation on these serum bead uptake assays was assessed by heating the serum to 56°C for 30 min prior to performing the assay.

Microscopy was performed on a subset of samples. The cells were re-suspended in PBS and smeared onto a glass slide and air dried with protection from light. A glass cover slip was applied onto the slide with FluorSave™ reagent solution. Light microscope images of latex bead uptake were taken on the Leica DM light microscope.

### Monocyte migration assays

Monocyte migration assays were performed using Neuro Probe ChemoTx® chemotaxis system 96 well-plates with 5 μm pores (http://www.neuroprobe.com/product/chemo_tx/). 29 μl of medium (RPMI (Life Technologies) and 10% FCS) with or without the chemoattractant CCL2 (100 ng/ml; Peprotech) was added to the appropriate lower wells of the plate in triplicate. 50 μl of monocyte suspension (2 × 10^5^ monocytes per 50 μl RPMI and 10% FCS) was added to the top of the filter above each well and plates were incubated at 37°C with 5% CO_2_ for 2 h. Cell migration into the lower wells was counted using a haemocytometer. The percentages of monocytes that migrated with and without CCL2 and the percentage increase in monocyte migration with CCL2 were calculated.

### Monocyte cytokine secretion assays

CD14^+^ monocytes were re-suspended in RPMI and 10% FCS at a concentration of 1 × 10^6^ cells per ml. 1 ml cell suspension was added per well into a 24 well-culture plate with and without the potent monocyte stimulant bacterial lipopolysaccharide (LPS) from Escherichia Coli O111:B4 (1 ng/ml; Sigma). Cells were cultured for 24 h at 37°C and 5% CO_2_. Post-culture supernatants were separated by centrifugation at 350 g for 5 min and stored at −80°C.

Cytokines were measured using a Mesoscale Discovery (MSD) platform V-Plex Pro-inflammatory panel 1 electrochemiluminescence assay (IFN-γ, IL-1β, IL-2, IL-4, IL-6, IL-8, IL-10, IL-12p70, IL-13, and TNF-α). The assays were run according to the manufacturer's instructions. Supernatant samples were diluted 1:10 in the appropriate buffer and assayed in duplicate.

### Statistical analysis

Between-group comparisons (PD vs. matched controls) were performed using paired *T*-tests (IBM SPSS statistics version 25). Experimental outliers with values >3 standard deviations (SD) above or below the mean were excluded prior to analysis. Values which were unpaired for any reason (e.g., outlier exclusion or cell unavailability for assay completion) were automatically excluded during the statistical analysis of paired *T*-tests. As analyses were always performed as a comparison between paired PD and control pairs, the exclusion of unpaired values did not influence sample matching.

## Results

### Participant demographics

41 PD patients and 41 age and gender matched controls were recruited in total. Demographic and clinical characteristics of the cohort are shown in Table 1. Subsets of samples were used for different functional assays depending on cell availability.

**Table 1:** Demographics of overall cohort.

**Variable**	**Patients**	**Paired controls**	***p***
Number (*n*)	41	41	
Age (years)	68.4 ± 6.3	68.1 ± 5.6	0.784
Gender (% male)	68.3	68.3	0.594
Disease duration (years)	4.2 ± 1.1	
MDS-UPDRS motor score	35.2 ± 12.3	
Equivalent Levodopa dose	591.5 ± 292.9	
ACE-R score	92.9 ± 8.2	

*Values indicate Mean ± SD (Standard deviation); UPDRS, Unified Parkinson's Disease Rating Scale; ACE-R, Addenbrooke's Cognitive Examination (Revised)*.

### Monocyte bead phagocytosis

The latex bead phagocytosis assay was performed using both standard medium (RPMI and 10% FCS) and freshly extracted autologous serum. While autologous serum was used to partially simulate the *in-vivo* intravascular environment, the standard medium represented assessment of cell-intrinsic phagocytic ability under standard culture conditions. A significantly higher percentage of monocytes phagocytosed latex beads in the presence of serum compared to standard medium (Figure 1), as would be expected due to the increased presence of opsonizing factors, such as antibodies and complement in autologous serum (23–25).

Paired analysis indicated no differences in measures of bead phagocytosis in standard medium between patients and controls (Figure 1). However, with autologous serum, the bead positive monocyte MFI ratio in patient monocytes was significantly higher than in controls (*p* = 0.0286; Figure 1), indicating increased uptake in PD bead positive monocytes compared to controls. Patients also tended to have a higher overall serum bead positive monocyte percentage, but this did not reach statistical significance (Figure 1). There were no statistically significant correlations between the uptake measures and clinical data, including the UPDRS motor score, in this cohort.

The influence of serum factors on uptake was subsequently examined in separate smaller *ad hoc* groups of patients and controls. These groups included patients of later disease stage compared to the original study, who otherwise fulfilled similar criteria. The effect of swapping PD and control serum with their paired monocytes was examined in 6 age and gender matched PD and control pairs [age- (mean ± standard deviation (SD)) PD 72.67 ± 2.34, controls 68.00 ± 6.89 (*p* = 0.148); gender 83.3% male (PD and controls); PD disease duration (mean ± SD) 7.35 ± 2.94]. The autologous serum bead positive monocyte percentage was significantly higher in PD patients compared to controls in this cohort (*p* = 0.0002; Supplementary Figure 2), but this difference was no longer apparent when phagocytosis was measured in the swapped, non-autologous serum condition (*p* = 0.6541). Thus, the origin of the serum appeared to account for the PD-control difference in monocyte phagocytosis (bead positive monocyte percentage) seen with autologous serum (Supplementary Figure 2).

Serum complement inactivation by heat treatment was examined in PD (*n* = 6) and controls (*n* = 5) [age (mean ± SD) PD 72.0 ± 2.44, controls 66.25 ± 8.18 (*p* = 0.135); gender PD 83.3% male, controls 80% male; disease duration PD 7.50 ± 2.69]. Serum heat inactivation resulted in significant decreases in autologous serum-based monocyte bead uptake in both PD and controls [bead positive monocyte percentage (PD *p* = 0.0001; controls *p* = 0.006); bead positive monocyte MFI ratio (PD *p* = 0.0034; controls *p* = 0.0163)], indicating that it had a significant effect on the overall level of phagocytosis (Supplementary Figure 3). Bead positive monocyte uptake was significantly higher in PD patients compared to paired controls (*p* = 0.0087) in this cohort, but this significant difference was lost post-heat inactivation (*p* = 0.0558; Supplementary Figure 3).

### Monocyte migration

**Figure 2:**
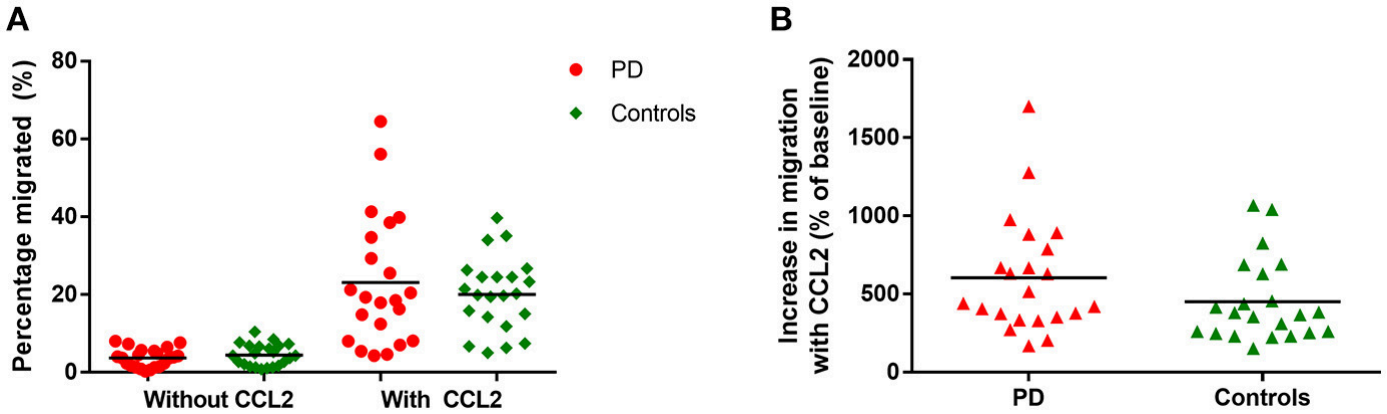
**(A)** Graph showing percentage monocytes migrated in all PD patients and controls, with and without the presence of CCL2 as a chemoattractant. PD = 22, Controls = 22. **(B)** Graphs showing percentage increase in monocyte migration with CCL2 in all PD and controls.

There were no significant differences in monocyte migration between this PD cohort and controls overall with or without the presence of CCL2 (Figure 2A). Monocyte trans-well migration levels were low in the baseline condition, but increased significantly, as expected, in response to CCL2. However, there was no difference in the magnitude of this response between PD and controls (Figure 2B).

There were no significant differences between patients and controls in the surface expression of the migration-associated surface markers CCR2 and CX3CR1 (Supplementary Figure 4). Migration measures also did not demonstrate any statistically significant relationships with clinical data, including the UPDRS motor score.

### Monocyte cytokine secretion

Cytokine secretion in the unstimulated condition was low for most cytokines and only IL-1β, IL-6, IL-8, IL-10, and TNF-α had measurable results in >75% of participants. There were no significant differences between paired patients and controls (Table 2).

**Table 2:** Summary of unstimulated and LPS stimulated monocyte supernatant cytokine results.

**Cytokine**	**Cytokine concentration (pg/ml)** ± **SD**					
	**Unstimulated**			**LPS Stimulated**		
	**Patients** **(*n* = 20)**	**Paired controls** **(*n* = 22)**	***p***	**Patients** **(*n* = 21)**	**Paired controls** **(*n* = 23)**	***p***
IFN-γ	X	X		46.09 ± 33.06	41.08 ± 35.00	0.764
IL-1β	42.74 ± 72.76	115.18 ± 232.63	0.176	771.38 ± 469.57	819.50 ± 500.82	0.222
IL-2	X	X		17.28 ± 21.16	14.37 ± 15.05	0.604
IL-4	X	X		4.87 ± 3.18	12.08 ± 16.68	0.082
IL-6	691.64 ± 2,027.38	1,060.22 ± 2,606.12	0.329	9,566.62 ± 4,558.47	10,024.70 ± 5,594.38	0.825
IL-8	28,776.83 ± 37,982.80	27,404.75 ± 39,478.76	0.591	126,025.80 ± 129,595.00	146,257.96 ± 166,969.57	0.794
IL-10	42.52 ± 104.54	63.59 ± 127.92	0.379	336.90 ± 235.92	327.90 ± 175.87	0.836
IL-12p70	X	X		22.78 ± 13.19	19.01 ± 11.05	0.605
IL-13	X	X		62.14 ± 51.80	50.93 ± 37.86	0.286
TNF-α	55.22 ± 123.60	215.93 ± 457.47	0.160	971.69 ± 612.36	962.76 ± 393.42	0.754

*All results represent mean cytokine concentration (pg/ml) ± standard deviation (SD). p-values indicate significance on paired t-tests between matched PD and control pairs. X indicates concentration above detection threshold in <75% of participants*.

With LPS stimulation for 24 h, measurable concentrations of all 10 cytokines were present in the supernatant in >75% of participants. However, there were no statistically significant differences between paired patients and controls overall in this cohort (Table 2). There were also no statistically significant relationships between cytokine secretion in either condition and clinical measures.

## Discussion

The findings demonstrate significantly increased monocyte phagocytic capacity in an autologous serum environment in PD, but no significant differences in standard medium-based monocyte phagocytosis, monocyte migration or cytokine secretion, compared to age and gender matched parallel processed controls, in this cohort.

The use of the participant's own serum as an environment for the uptake assays attempted to more faithfully replicate the intravascular *in-vivo* environment. The presence of serum increased bead phagocytosis overall, compared to standard medium in both PD and controls, consistent with the presence of opsonising factors, such as antibodies and complement in the serum (25, 26). Complement inactivation through heat treatment had a profound negative effect on bead uptake in both PD and controls, indicating an important contribution of complement to serum-based monocyte phagocytosis (Supplementary Figure 2). Interestingly, derivatives of complement factors, such as C3, have been found to be higher in PD serum (27) compared to controls and may contribute to the differences seen. We did not specifically investigate the effect of serum antibodies in this study, but they may also play an important role. Further studies including antibody depletion and Fc receptor blocking and measurement of serum complement and antibody levels will be required to fully determine the factors responsible for the serum assay differences observed.

Our data suggests that serum components in disease are an important factor in determining the differential phagocytosis observed between PD and controls. When the environmental conditions are equal (standard medium) there is no significant difference between PD and control monocyte phagocytosis, whereas an autologous serum environment brings out a difference. This indicates a strong influence from cell extrinsic factors rather than cell-intrinsic factors in bringing about the PD-control difference in phagocytosis observed in this study. The data also suggests that swapping patient and control serum with their paired monocytes removes this PD-control difference, further supporting the hypothesis that external disease related serum-based factors critically influence monocyte phagocytic behavior.

In contrast to our study, previous studies investigating monocyte phagocytosis in PD using standard medium conditions have found a relative impairment in PD compared to controls in smaller numbers of participants (2, 3). The standard medium bead phagocytosis assays in the current study, which would be most similar to those previous studies (3) did not show a similar significant impairment. However, the assays used in the previous studies utilized different types of medium, different types and sizes of uptake particles (different concentrations of 1 μm beads, fluorescent red blood cells or zymosan particles) and longer assay periods (up to 4 h) and were also performed on relatively later stage PD patients (2, 3), which may have contributed to the differences in outcome. Shorter incubation periods used in the current study may have also resulted in persistence of elements of influence from the *in-vivo* environment, which may have affected the results.

Previous studies have also shown the presence of differences in monocyte surface markers and other cell-intrinsic factors in PD vs. controls (3, 28). It is possible that intrinsic monocyte differences may enhance or enable the effects of serum and the overall phagocytosis level is likely to be influenced by a combination and/or interaction of intrinsic and extrinsic factors. Further studies investigating these factors in larger numbers of PD and control pairs will be important to confirm and extend these findings.

This was also the first study to assess monocyte migration behavior in PD, and we found no significant overall differences in standard medium based migration when compared to controls in this cohort. One previous study has found increased expression of the chemokine receptor CCR2 in PD monocytes compared to controls (28), but our data did not replicate this finding. Alpha-synuclein monomers and oligomers have been shown to have monocyte and neutrophil chemoattractant properties (18), but this was not assessed in the current study. Considering the phagocytosis differences seen with autologous serum, it would be interesting to also assess monocyte migration in autologous serum, to investigate any differences which may be revealed in this more *in-vivo* relevant environment. However, these assays would need to be controlled for serum CCL2 and other chemokine levels, which may be potential confounding factors.

The current study showed no significant differences in standard medium based monocyte cytokine secretion between PD and controls. Previous studies using smaller sample sizes have reported inconsistent results, with increased (3) or partially decreased/unchanged (4) cytokine secretion by PD monocytes with LPS stimulation compared to controls. The largest of these (21 PD, 8 controls), reported no significant differences in monocyte IL-1β, IL-6, IL-8, and IL-10 production, in keeping with our study, but found a decrease in TNF-α secretion by PD monocytes (4). Elevated cytokine production by PBMCs has also been reported in PD compared to controls (29), suggesting that other immune cell types may also be involved in the mediation of an increased inflammatory response in PD patients.

As with migration, monocyte cytokine secretion could also be assessed in autologous serum, but measurement of supernatant cytokine levels would require controlling for serum intrinsic cytokine levels. However, measurement of monocyte intracellular cytokine levels using intracellular staining and flow cytometry and cytokine gene expression may be alternative methods of assessing cytokine secretion in the serum environment.

The functional assays were performed on positively selected CD14^+^ monocytes and the overall functional status of total monocytes will be influenced by the relative proportions of the different monocyte subsets in PD and controls, with the predominant classical monocytes (~60–80% of total monocytes), which have been found to be higher in PD, likely to have the most influence on functions, such as phagocytosis (3). However, this would not explain the presence of significant differences in phagocytosis in autologous serum, but not in standard medium. CD14^+^ selection may also lead to the relative loss of CD14 low non-classical monocytes, which may subsequently influence the magnitude of any potential differences in non-classical predominant cytokine secretion. Nevertheless, a previous study demonstrating significant differences in monocyte secretion also used CD14^+^ selection, suggesting that this may not be a major factor (3). In general, the goal of the study was to obtain a global overview of the status of total monocyte function in PD compared to controls, while accepting that a variety of factors will likely be contributing toward the overall picture.

It is possible that the use of CD14 positive magnetic bead selection may have affected monocyte behavior (30). However, both patient and control samples underwent the same process of monocyte extraction and previous studies have also used this method of monocyte isolation with differing results (3), hence it is unlikely to be a major factor influencing differences between PD and controls.

Most of the PD patients involved in this study were on levodopa or dopamine agonist medications, and dopamine has been reported to have effects on immune cells, including augmentation of T follicular helper cell-B lymphocyte interactions in germinal centers (31) and of monocyte functions, such as migration (32). However, we found no significant differences in monocyte migration behavior in PD when compared to controls and this coupled to the finding of no significant correlations between monocyte uptake measures or other functions and levodopa equivalent dose in this study, suggests that it is unlikely that these drugs could explain the differences in behavior that we report. Dietary, circadian and other medication factors may also potentially influence monocyte function and serum. In this study all patients and controls had samples taken within the same time period in the morning and would have been expected to have had breakfast and their regular medication prior to their visit. Further work will require additional assessment of these assays in dopamine medication naïve earlier stage PD patients and paired controls, prior to food and medication intake in the morning.

It is possible that clinical subgroups of patients, with differing levels of risk factors for cognitive and motor progression (33, 34) may have differential differences compared to paired controls and that potential differences are masked with the use of overall combined analysis. This study did not have sufficient numbers for each assay, to enable subgroup analysis. Future studies will need to repeat these assays in larger numbers of different clinical subgroups of patients to determine any underlying differential changes seen only within specific clinical subgroups. The uptake assays investigating serum factors provide some indication that serum-based uptake is higher in PD even at later stages of disease. Thus, it will be important to assess monocyte function in larger cohorts at different stages of PD, as well as longitudinally, in order to identify any changes correlated with disease severity and duration, which may be useful as potential biomarkers.

In conclusion, we have investigated a range of monocyte functions in early-moderate PD compared to age and gender matched controls and demonstrated an increased capacity for bead phagocytosis in PD which appears to be mostly driven by cell-extrinsic factors in PD serum. This adds to existing evidence implicating changes in innate immune function in PD, but further work will be required to investigate what serum factors are important, as well as the physiological and clinical relevance of these findings, particularly in relation to the disease process and the alpha-synuclein pathology in PD.

## Author contributions

RW, CW-G, and DK-V designed the study and planned the assays. RW, CW-G, KS, and SH carried out the study. MC, RAF, JJ, and DK-V advised on the assay protocols, interpretation and analysis. RW prepared the first draft of the manuscript. CW-G, DK-V, RB, KS, JJ, MC, RAF, and SH reviewed the manuscript.

### Conflict of interest statement

The authors declare that the research was conducted in the absence of any commercial or financial relationships that could be construed as a potential conflict of interest.
