# Supplementary material for: Monocyte Function in Parkinson's Disease and the Impact of Autologous Serum on Phagocytosis
Source: Front Neurol. 2018 Oct 16;9:870. doi: 10.3389/fneur.2018.00870 (PMC6198066; doi:10.3389/fneur.2018.00870)
Supplement: Supplementary file 1 [file Data_Sheet_1.docx]

**Monocyte Function in Parkinson’s Disease and the Impact of Autologous Serum on Phagocytosis**

R.S. Wijeyekoon, D. Kronenberg-Versteeg, K. M. Scott, S. Hayat, J. L. Jones, M. R. Clatworthy, R. A. Floto, R. A. Barker, C. H. Williams-Gray.

**SUPPLEMENTARY DATA**


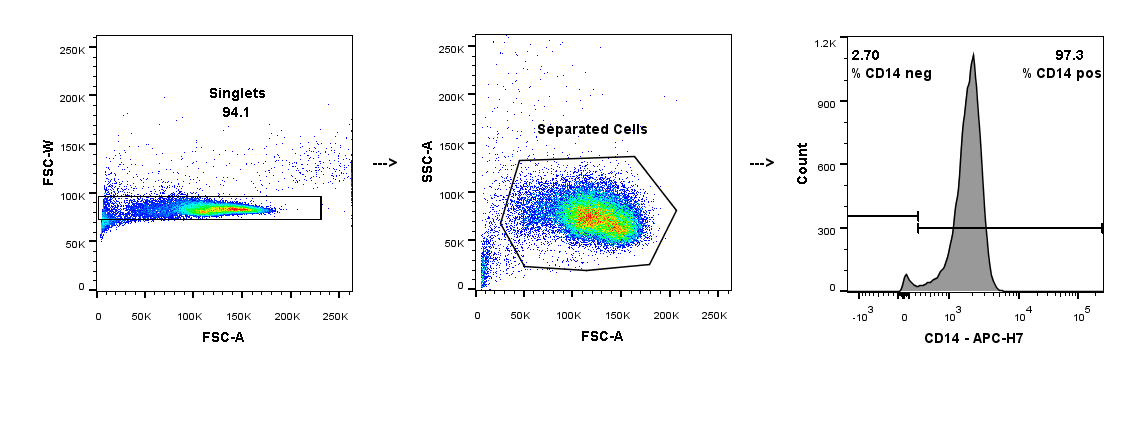


***Supplementary Figure 1A*** *- Flow cytometry plots showing gating of CD14 magnetic bead separated monocytes, with staining for CD14-APC-H7. CD14+ monocyte cell purity post separation was overall >97%. FSC-A=Forward scatter –Area; FSC-W = Forward scatter-width; SSC-A= Side scatter-Area*


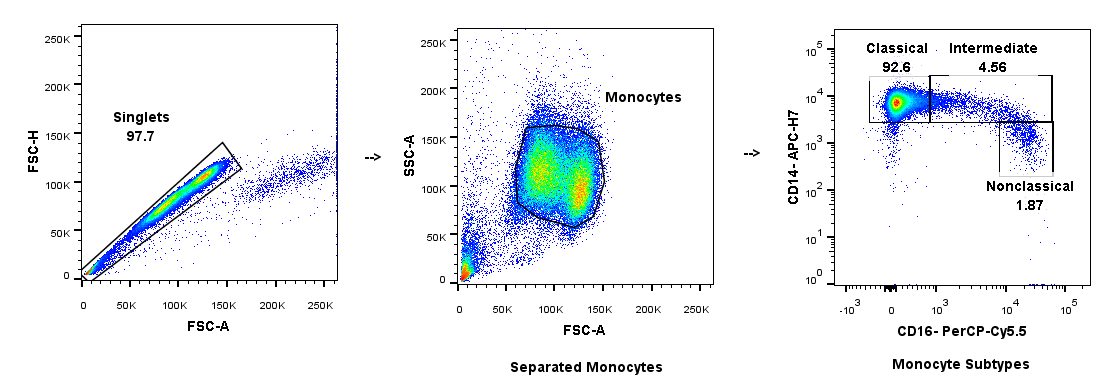


***Supplementary Figure 1B –*** *Post CD14+ magnetic bead separated monocytes. CD14/CD16 staining indicates the presence of Classical, Intermediate and Non-classical monocytes post separation. FSC-A=Forward scatter –Area; FSC-H = Forward scatter-height; SSC-A= Side scatter-Area*


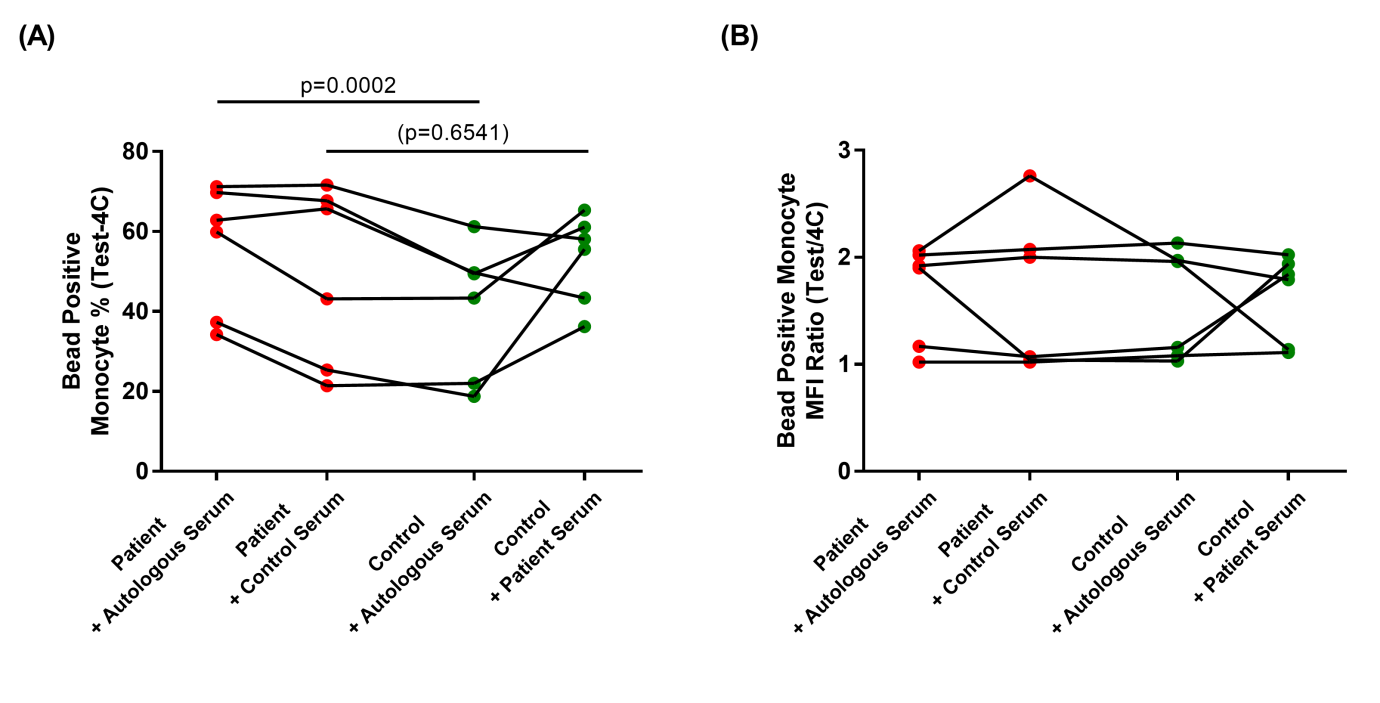


***Supplementary Figure 2***

*Effect of swapping PD and Control serum with their corresponding monocytes on phagocytosis (n=6 pairs). (A) Bead positive monocyte percentage; (B) Bead positive monocyte MFI ratio.*


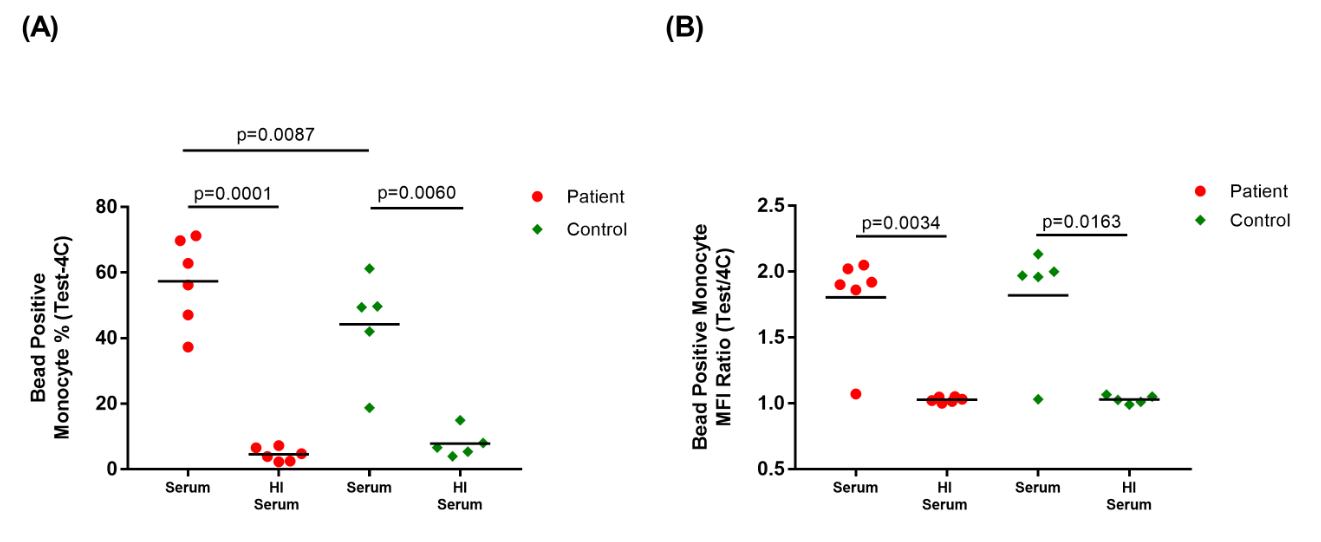


***Supplementary Figure 3***

*Sample phagocytosis assays in autologous untreated serum, and autologous heat inactivated (HI) serum (PD=6, Control=5) showing effect of heat inactivation on phagocytosis.*

*(A) Bead positive monocyte percentage; (B) Bead positive monocyte MFI ratio.*


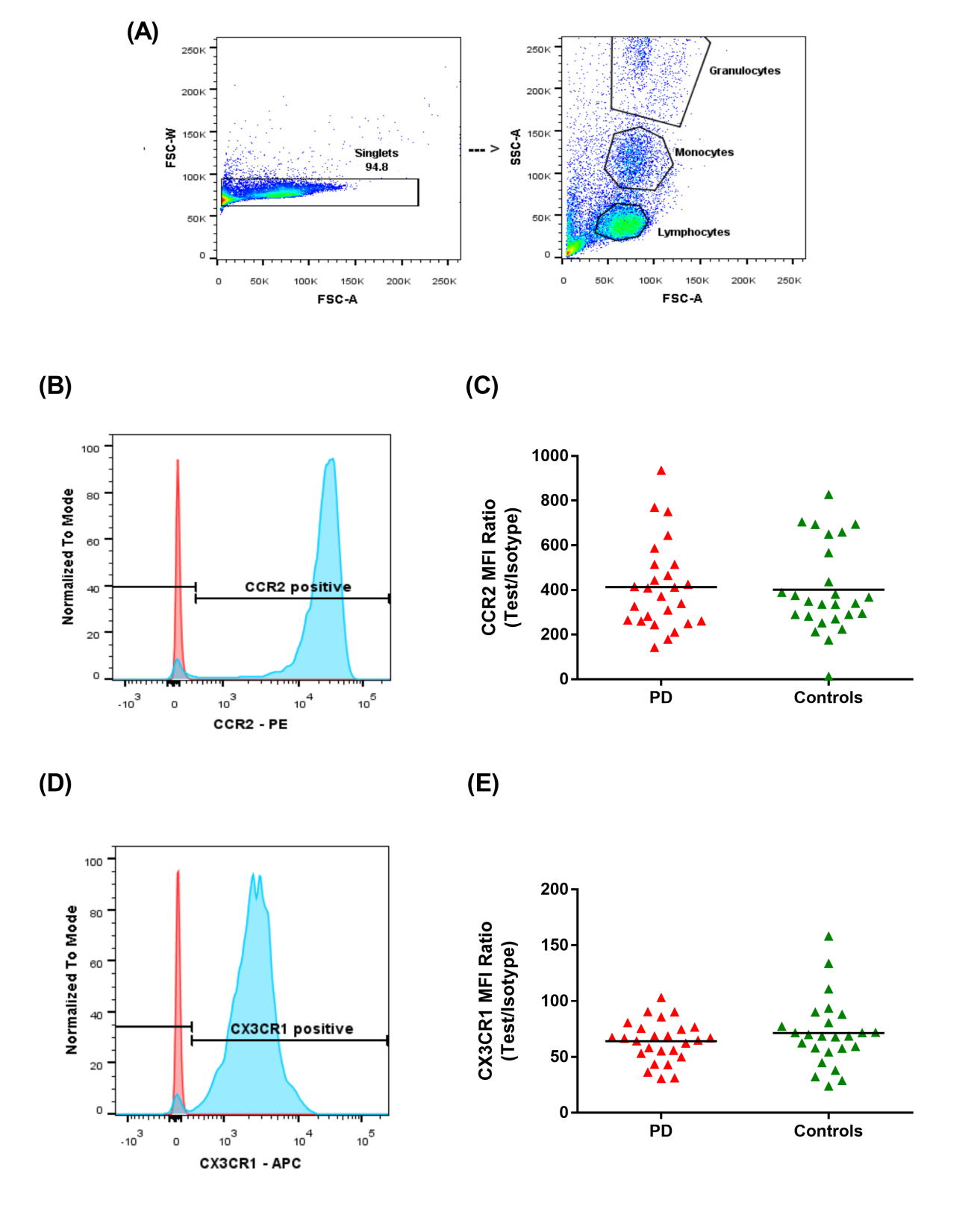


***Supplementary Figure 4 –*** *(A) Example of the gating strategy for monocytes within PBMCs. Singlets were identified by plotting Forward Scatter-Area (FSC-A) versus Forward Scatter-Width (FSC-W). Monocyte, lymphocytes and granulocytes were distinguished using FSC-A (size) and Side Scatter-Area (SSC-A) (granularity).*

*(B) and (C) CCR2 expression on total monocytes (B) Histogram indicating CCR2-PE staining (blue) compared to isotype (red). (C) Total monocyte median fluorescence intensity (MFI) ratio (Test/Isotype) for CCR2 in PD (n=26) and Controls (n=26).*

*(D) and (E) CX3CR1 expression on total monocytes. (D) Histogram indicating CX3CR1-APC staining (blue) compared to isotype (red). (E) Total monocyte median fluorescence intensity (MFI) ratio (Test/Isotype) for CX3CR1 in PD (n=26) and Controls (n=25).*
